# Multi-Agent Adaptive Traffic Signal Control Based on Q-Learning and Speed Transition Matrices

**DOI:** 10.3390/s25237327

**Published:** 2025-12-02

**Authors:** Željko Majstorović, Edouard Ivanjko, Tonči Carić, Mladen Miletić

**Affiliations:** University of Zagreb, Faculty of Transport and Traffic Sciences, Vukelićeva Street 4, 10000 Zagreb, Croatia; zeljko.majstorovic@fpz.unizg.hr (Ž.M.); tonci.caric@fpz.unizg.hr (T.C.); mladen.miletic@fpz.unizg.hr (M.M.)

**Keywords:** multi-agent, speed transition matrix, Q-Learning, adaptive traffic signal control, connected vehicles

## Abstract

Advancements in technology and the emergence of vehicle-to-everything communication encourage new research approaches. Continuously sharing data through the onboard unit, connected vehicles (CVs) have proven to be a valuable source of real-time microscopic traffic data. Utilizing CVs as mobile sensors is a key driver for traffic safety improvement and increasing the effective operative road capacity. Data obtained from CVs can be effectively processed using speed transition matrices (STMs) while preserving spatial and temporal characteristics. This research proposes a new approach to adaptive traffic signal control utilizing STMs and a cooperative multi-agent learning system for the environment of CVs. To confirm its effectiveness, the concept is tested in a simulated environment of an intersection network, comparing different CVs’ penetration rates and cooperation coefficients between agents.

## 1. Introduction

The emergence of connected vehicles (CVs) has generated new technological needs, as well as impacts in the social and economic fields. A CV is considered any vehicle equipped with an on-board unit able to communicate with the environment. Market reports show that nearly 45% of new vehicles sold in 2020 were equipped with some form of connectivity, and by 2030, almost all vehicles are expected to be connected. This trend is generated by consumers’ demand for infotainment systems, advanced driver assistance systems (ADAS), and vehicle-to-everything (V2X) communication technologies [1]. The gradual emergence of CVs and their coexistence with human-driven vehicles generate a new type of traffic flow, so-called mixed traffic flows, consisting of classic human-driven vehicles and connected autonomous vehicles (CAVs) [2]. Thanks to their advanced sensing capabilities, CVs act as mobile sensors, providing real-time data, as compared to conventional sensors that cover only a particular section of the road. Thus, CVs are superior to current traffic sensor technology. Unlike, for example, cameras, CVs are not constrained by line of sight, and the collecting of data at the microscopic level enables new approaches to traffic analysis and traffic signal control.

The most common machine learning (ML) methodology for traffic signal control (TSC) is reinforcement learning (RL) due to a relatively simple algorithmic structure based on a trial and error learning process [3]. Knowledge is stored in the form of a table, known as state–action pairs. That knowledge is built over time with an algorithm visiting each state-action pair, calculating a so-called Q-value based on the received reward. Deep reinforcement learning (DRL) is a subcategory of RL where the table with the Q-values for each state–action pair is replaced with a deep neural network (DNN), where the DNN predicts Q-values for selected actions in a given state.

DRL is suitable for complex problems with a large input space. However, the convergence is slower, especially in the early learning stage, because the DNN has to learn states and rewards simultaneously. Thus, a DRL approach can be more time consuming in certain use cases compared to RL, which populates the Q-table with calculated values.

Furthermore, interpretable RL has also gained more popularity. DNNs learn abstract representations of data layer by layer, which lack a direct correspondence in the original input space, making it difficult to interpret the relationships between these abstract features, input data, and output results. Consequently, the complexity of a DNN architecture makes it challenging to intuitively understand the role and contribution of each neuron and parameter during the learning process, thereby rendering the decision-making and reasoning logic opaque [4].

This research is an extension of previous research that was focused to the applicability of speed transition matrices (STMs) for traffic state estimation in an environment of mixed traffic flow. The research described in [5] analyzes traffic state estimation using STMs on an isolated intersection, while the research described in [6] analyzes the CVs’ penetration rate impact on the quality of the information. Obtained results indicated that at a 20% CV penetration rate, CVs already provide sufficient information for the STM-based traffic state estimation. Research results on RL-based TSC on an isolated intersection that uses STM for traffic state estimation were presented in [7]. Encouraged by the obtained results, the research is extended from isolated intersection control to the control of a small network of intersections, and from a single-agent to a multi-agent RL (MARL) system.

The main motivation for this research stems from the growing prevalence of CV and the availability of a wealth of real-time traffic data at the microscopic level, which enable new advances in traffic data exploitation. One can expect more reliable state estimation results with more in-depth situation information (free flow, various congestion levels, and incident detection) for intersection control when microscopic traffic data are used. However, the problem of real-time processing of a large amount of data occurs. In the case of CVs, every vehicle generates a data stream, compared to data streams of a small number of classic traffic flow sensors, like inductive loops, cameras, or radars. Additionally, CV data provide information about the spatiotemporal traffic state, which should be utilized to improve adaptive traffic signal control (ATSC). STMs can cope with these challenges, as first results proved in our previous research [5,6,7,8]. However, the open question remains regarding the control of a network of connected intersections.

A prerequisite for the operation of ATSC systems is the ability to assess the intersection state in a timely manner so that the control system can adapt to the new intersection state. Driven by the results of our research, we see STMs as a potential solution for assessing the state of a network of intersections, which is a scientific contribution to existing solutions. Thus, the main contribution of this paper is the application of the STM for the traffic state estimation on the small network of intersections, and the development of a MARL-based ATSC applying STM-based state estimation for the network of intersections, which has not been addressed so far. Compared to the previous approach of ATSC on an isolated intersection [7], this paper introduces ATSC on multiple intersections, where each intersection is controlled by one agent. Additional intersections add complexity to the control system, where agents can have negative impacts on neighboring intersection (e.g. offloading traffic demand to the neighboring intersection), which will indicate how well the proposed STM-based approach can detect the state of the traffic network. Furthermore, previous research relied on a 100% penetration rate of CVs, while this research includes penetration rate analysis ranging from 10 to 100%. However, it is assumed that vehicle communication is reliable. Thus, the issues related to V2X communication reliability or sensor failures are beyond the scope of this paper.

The rest of this paper is organized as follows. In Section 2, a related work overview is provided. Section 3 describes the methodology for CV data complexity reduction. A description of the proposed MARL approach is provided in Section 4, followed by results in Section 5. In Section 6, the obtained results are discussed, and in Section 7, the conclusion is provided.

## 2. Related Work

Modern TSC increasingly relies on systems capable of adapting to real-time conditions. Two key research areas supporting this development are ATSC and intersection state estimation. In such cases, adaptive control strategies aim to optimize signal timings based on current traffic demands, while state estimation methods focus on accurately capturing the dynamic conditions at intersections using real-time sensor data and predictive models. Related work can be divided into the following categories: ATSC and intersection state estimation. The following subsections provide an overview of relevant research papers in each of these areas, emphasizing their contributions.

### 2.1. Adaptive Traffic Signal Control

ATSC enables a TSC system to adapt to current or expected traffic demand, adjusting the signal program timing accordingly. It was first proposed in the 1960s, where the first research and implementations included ATSC systems such as SCOOT, SCATS, and UTOPIA [9,10,11]. ATSC systems adapt to traffic fluctuations with a fast response to newly emerged situations. While operating, they are solving signal program optimization problems regarding selected optimization objectives (e.g., intersection throughput maximization, delay minimization, or giving priority to public transportation or emergency vehicles). Regarding ATSC, recent approaches include applications of ML and the use of CVs as data sources to achieve improvements regarding signalized intersection performance.

In [12], the authors relied on vehicle probe data to achieve coordinated control of intersections of arterial roads. Instead of relying on common parameters such as travel time, queue length, or delay, the authors observed the number of non-stopped vehicles in the total number of vehicles as a traffic state indicator. Developing RL-based ATSC in [13], the authors combined signal plan optimization and CAV speed guidance. An RL-based system optimizes the signal plan to minimize the total queue length while allowing the CAVs to adjust their speed based on an existing fixed traffic signal control (FTSC) system to decrease the total delay. Obtained results of average delay and queue length indicated that the proposed method outperforms FTSC as well as conventional ATSC, particularly in saturated and oversaturated conditions. Conventional ATSC is based on the guidelines of the Federal Highway Administration (FHWA) [14]. Some recent approaches even utilize digital twins to simulate different traffic scenarios and evaluate appropriate traffic control strategies [15,16]. However, estimating the state of the intersection remains a crucial step in the implementation of ATSC [17].

### 2.2. Intersection State Estimation

The estimated process state depicts the intersection level of service, throughput, average queue length, (average) vehicle speed, and so on. Depending on the estimated state, changes of the traffic signal program can be made. Thus, the signal phase duration or sequence can be adapted. State estimation assessment is made based on collected data; thus, strict categorization of research papers is often not feasible since the authors combine various data sources and methods. In research papers, three main method types can be distinguished: (i) model-driven, (ii) data-driven, and (iii) streaming-data-driven methods [18]. Model-driven methods are based on models that represent physical traffic flows. Such models can explain estimation inaccuracy thanks to their explanatory characteristics. A poorly calibrated traffic flow model can affect the performance of the estimation method. Data-driven methods rely on historical data, which are mostly collected by GPS-equipped delivery or taxi vehicles. Relying on historical data, data-driven methods are prone to failure in cases when an unexpected event occurs or when traffic trends change over time. These methods are usually coupled with ML techniques to identify traffic state based on features in historical data. The authors in [19] used GPS data clustering to detect turn-level congestion. Relying on the clustering, the proposed method identified spatiotemporal characteristics of congestion at the turn level. In [20], the authors proposed a novel method for traffic state estimation based on STMs. The obtained results indicated that the proposed method is capable for traffic state estimation on macro- and micro-locations in the city area.

Streaming-data-driven methods have gained interest in recent research since they do not require historical data. Relying on streaming data and weak assumptions for future prediction, these methods are robust to unpredictable events. For accurate estimation, these methods require large amounts of real-time data, which is a drawback of such methods. These methods include various data sources such us video detectors, ultrasonic sensors, infrared sensors, acoustic sensors (sonars), and others [21]. Relying on a single data source may not be effective; thus, data fusion from various sources to gain robustness is one of the approaches used. Utilizing data from different sources, the authors of [22] fused data from three different sources: stationary flow sensors, turning ratios, and floating car data. The research [23] is an example of using advanced sensor technology as a data source, exploring the variation of state space patterns for different traffic scenarios. The authors established a relationship between the traffic demand and the actual traffic states and applied a trained DRL model to new traffic scenarios without additional training. In [24], the authors addressed the issue of mutual influence of neighboring agents in a control system managing a small network of intersections, since the actions of one agent (signal program change on one intersection) can influence the performance of neighboring agents. For the traffic state estimation, growing neural gas (GNG) was used. Results indicated that the most effective ATSC strategy was centralized state estimation using GNG combined with decentralized agent execution. This approach with centralized data processing ensured accurate state estimation while maintaining flexibility and scalability.

## 3. CV Data Complexity Reduction

This section outlines the methodology for reducing the complexity of gathered real-time CV data. Data complexity reduction is a crucial step in the traffic state estimation process where the goal is to simplify the data space while preserving as much information as possible. The authors of [25] proposed a DRL-based TSC system for an isolated intersection. In the proposed approach, the state is represented with the following traffic parameters: the number of vehicles per lane at time step, the queue length per lane at time step, and waiting time for all vehicles on the lane. The data are represented as a vector. This example shows how even at an isolated intersection, state representation parameters can be demanding. Applying such an approach to multiple intersections would be considerably more complex.

### 3.1. Speed Transition Matrix

The STM traffic data modeling method is adopted from [20]. The STM is a matrix that represents the speed change when vehicles are traveling between two consecutive road segments in the observed time period ∆t. Two consecutive road segments make a single speed transition, where the first segment is considered as the origin and the second segment is considered as the destination. For each origin–destination pair, the harmonic mean speed is calculated and written in the STM at position (m,n), where *m* represents the speed at the origin, and *n* represents the speed at the destination segment. The expression used to describe the STM is as follows:

(1) $$X\left(∆t\right)=\;\left(\begin{array}{cccc} p_{\left(1,1\right)} & p_{\left(1,2\right)} & \ldots & p_{\left(1,n\right)}\\ p_{\left(2,1\right)} & \ddots & \; & \vdots \\ \vdots & \; & \ddots & \vdots \\ p_{\left(m,1\right)} & \ldots & \ldots & p_{\left(m,n\right)} \end{array}\right),$$

where $p_{\left(m,n\right)}$ denotes the resulting probability of the speed change between an origin (*m*) and destination (*n*) road segment at ∆t interval. These probabilities are obtained on top of all collected speed transition data measured by CVs. Thus, a large number of microscopic vehicle speed measurements can be processed in real time, preserving important intersection state information on all respective inbound roads.

**Figure 1:**
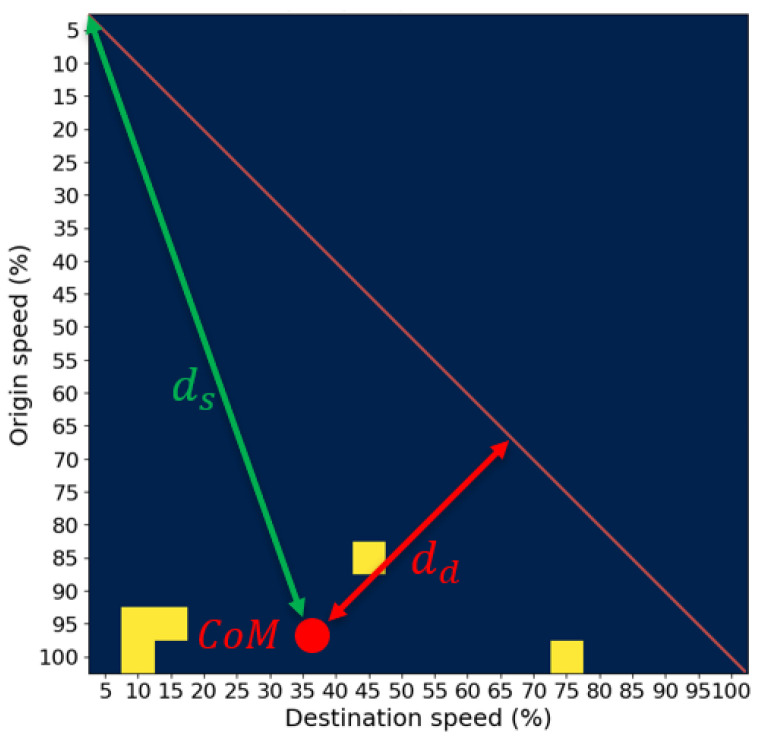
STM matrix with feature parameters $d_{s}$ and $d_{d}$.

One has to note that the data written in the matrix have their own center of mass (CoM) whose position depends on the processed speed data arrangement in the STM matrix. The position of $CoM_{j,k}$ in the STM matrix is described with two parameters: distance from the source $d_{s}$, and distance from the diagonal $d_{d}$. The distance $d_{s}$ is calculated as the Euclidean distance from $p_{0,0}$ to the $CoM_{j,k}$, and the distance $d_{d}$ is calculated as the Euclidean distance from the matrix’s diagonal to the $CoM_{j,k}$ as shown in Figure 1.

**Figure 2:**
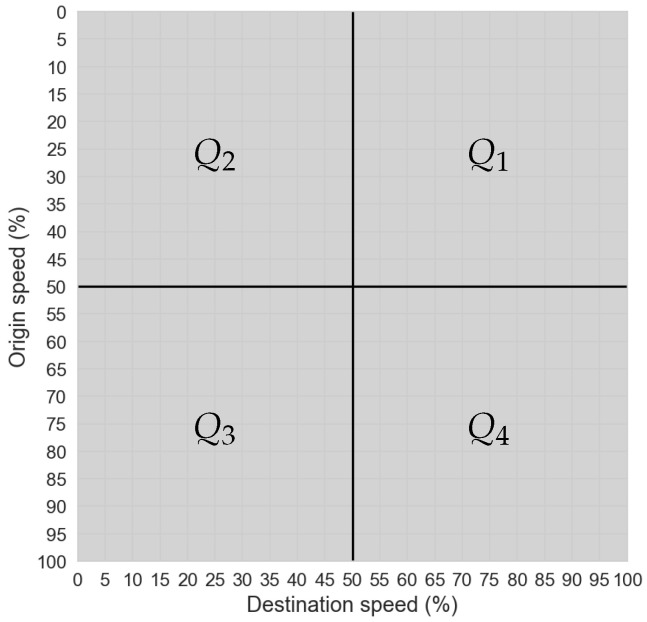
STM interpretation per quadrant.

The traffic state at the observed road segment can be interpreted depending on the respective CoM position in the matrix as shown in Figure 2. Each STM quadrant describes a certain state as follows:

- $Q_{1}$—vehicles are gaining speed because the vehicles have low origin speed and high destination speed;
- $Q_{2}$—vehicles are moving slowly because they have low origin speed and low destination speed;
- $Q_{3}$—vehicles are slowing down because they have high origin speed and low destination speed;
- $Q_{4}$—vehicles are moving freely because they have high origin speed and high destination speed.

Note that $d_{d}$ and $d_{s}$ are parameters that characterize the traffic state represented in the STM. Extracting these parameters allows the STM to be used not only for visualizing traffic conditions but also for broader analytical and operational traffic control applications such as TSC. The benefit of the STM is that the traffic state can be easily assessed by calculating the $d_{d}$ and $d_{s}$ parameters. Thus, just by visually observing the STM, one can have an idea of the traffic state on the observed road segment. Even at relatively low CV penetration rates of just 20%, the provided data were sufficient for the traffic state estimation using STMs, as observed in [6]. Here, one can determine the benefit of using the STMs, as the variability of vehicle speed transitions is automatically filtered out with the CoM-based interpretation approach. Such speed variability happens during signal phase changes and under different driving characteristics.

### 3.2. Self-Organizing Maps

A self-organizing map (SOM) is a type of neural network with only one layer that uses competitive training to arrange neuron weights to match the received input signal [26]. Neurons in an SOM are arranged in a grid with lateral connections between them. Weights in an SOM can be considered as coordinates in the input space. The primary principle of SOM operation is “winner takes all”, meaning that each input signal will be matched to the closest (winning) neuron, which will then be called the best matching unit (BMU). The distance between the input signal and each neuron is usually calculated with a Euclidean distance formula. After receiving the input signal, the weights of the BMU and other connected neurons will then be adjusted according to the following equation:

(2) $$W_{i}\left(t+1\right)=W_{i}\left(t\right)+\Theta \left(t\right)\alpha \left[X\left(t\right)-W_{i}\left(t\right)\right],$$

where $W_{i}\left(k+1\right)$ is the weight vector of neuron *i* at time step t+1; Θ(t) is the neighborhood function, which scales the update depending on the distance between neuron *i* and the BMU; α is the learning rate; and X(t) is the vector of the input signal in time step *t*. Common functions for the neighborhood function Θ are the Gaussian or Ricker wavelet [27]. Both functions decrease the weight change if the neuron is far from the BMU. In this paper, a Gaussian neighborhood function is used according to [27]:

(3) $$\Theta \left(t\right)=\exp\left(-\frac{\|W_{i}-W_{BMU}{\|}^{2}}{2\sigma (t)}\right),$$

where σ represents the width of the neighborhood, which decreases in time.

When SOM training is complete, the grid formed by neurons will preserve the topological structure of the original input data set, but in a lower dimensional space, since the formed grid is two-dimensional. For this reason, SOM is commonly used as a dimensionality reduction and clustering technique. After training, each point in the input space can be matched to its closest neuron, since each neuron will form a Voronoi cell from its position. In this paper, each neuron or group of closely placed neurons will represent a respective intersection traffic state needing a specific control action (i.e., signal program adaptation).

## 4. Multi-Agent Reinforcement Learning Approach

This section presents the implemented MARL approach used for this research, describing its structure and role in a small intersection traffic network optimization. The goal is to reduce congestion, which leads to reduced travel times, generally utilizing the Q-Learning algorithm.

### 4.1. Q-Learning

The Q-Learning algorithm modifies Q-values in the Q-matrix, representing state–action pairings when environmental conditions reach a specific state. It uses a feedback loop mechanism to evaluate the efficacy of an action in a specific state. The algorithm is stochastic, aiming to select the best potential action with the highest Q-value if it runs indefinitely. The Q-Learning execution produces the highest achievable Q-value for every state–action combination, ensuring every state has a highest Q-value indicating the optimal action within that specific state. The Q-value is updated when an agent chooses an action in a given state, as described in [28]:

(4) $$Q\left(s_{t},a_{t}\right)\leftarrow Q\left(s_{t},a_{t}\right)+\alpha \left[r_{t}+\gamma \underset{a^{\prime }\in A}{arg max}Q\left(s_{t+1},a^{\prime }\right)-Q\left(s_{t},a_{t}\right)\right],$$

where the *Q* value for each state (*s*)–action pair (*a*) at the time step *t* is denoted with $Q(s_{t},a_{t})$, and the reward at time step *t* is denoted with $r_{t}$. Depending on the actions selected, the reward will be positive or negative. In this research, the reward is always negative, and the algorithm seeks to maximize its reward, thereby minimizing the time-loss value. The discount factor γ quantifies the significance of future rewards in the following state, and α represents the learning rate that determines the rate at which the Q-Learning algorithm obtains new information and updates Q-values.

The exploration–exploitation trade-off during learning is modeled with the use of the ϵ-greedy selection policy [29]. Initially, the ϵ value is high to foster more exploration, thus enabling the agent to learn about its environment. At the later stages of learning the procedure, the value of ϵ is decreased, forcing the agent to exploit existing knowledge. To ensure a smooth transition from exploration to exploitation, the ϵ policy is modeled as the sigmoid function Equation (5):

(5) $$\epsilon =\left(\frac{a-b}{1+{\left(e^{x-\frac{c}{2}}\right)}^{d}}\right)+b,$$

where a=1 defines the amplitude, b=0.05 adjusts the *y*-offset, c=100 adjusts the *x*-offset, and d=0.15 adjusts the slope. The variable *x* is the input value of the number of the current simulation.

### 4.2. RL-STM-SOM Integration

The integration of the STM and SOM facilitates data complexity reduction. In the case of intersection control, one has to take into account that each intersection has four inbound roads, and each inbound road contains multiple road segments in which vehicle speed transitions were periodically recorded during FTSC operation to gather information about various intersection states. In our previous research paper [5], the applicability of the STM for estimating the state of an independent intersection was analyzed, and the results showed that the STM can detect congestion on the inbound roads of intersections. Such a result indicated that STM can be used for intersection state estimation in the case of independent intersection control.

Data collected on each speed transition are stored in the STM. Extracting STM features $d_{d}$ and $d_{s}$ for each intersection inbound road forms the input vector for the SOM as follows:

(6) $$v=\left[\begin{array}{c} x_{1}\\ x_{2}\\ \vdots \\ x_{n} \end{array}\right],x_{n}=\left[\begin{array}{c} d_{s_{1}},d_{d_{1}},\ldots ,d_{s_{n}},d_{d_{n}} \end{array}\right],$$

where *v* represents the input vector for training the SOM network. Each $x_{n}$ denotes a single observation of the traffic network, consisting of multiple STM features $d_{d}$ and $d_{s}$. The number of STM features depends on the number of observed speed transitions. After enough data are collected, the SOM network is trained offline.

After training, the SOM clusters similar traffic patterns, allowing the identification of traffic states within the network. The trained SOM network is sized as a 4×4 network, which is 16 neurons in total, where each neuron represents a possible state of the analyzed intersection. It should be noted that the intersection is operated using the FTSC approach, and traffic patterns emphasize a typical working day. The neural network size was determined through testing various configurations. If the network were smaller, for example 3×3, the reduced number of neurons would cause multiple states to map to the same neuron, reducing representation accuracy. Conversely, increasing the number of neurons can lead to some neurons remaining unused, representing no data cluster. The chosen size ensures that each neuron is activated at least once, providing a balanced trade-off between granularity and coverage.

**Figure 3:**
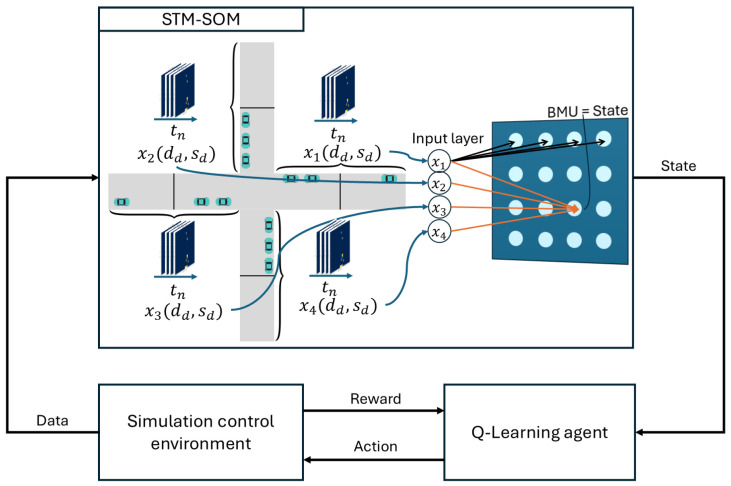
Schematic representation of the proposed RL–STM–SOM integration.

The STM–SOM integration is extended with the Q-Learning algorithm as shown in Figure 3. The STM–SOM integration reduces the complexity of the collected microscopic-level CV traffic data and identifies the state of the intersection, where a single state is defined for all intersection inbound roads. The input vector, which contains the data collected on observed inbound roads, is represented by one neuron (i.e, intersection state), which is further used as input for the Q-Learning algorithm. In our implementation, the Q-Learning algorithm can select from four predefined signal programs every five minutes. Four predefined signal programs are implemented at a real-world intersection. A five-minute interval was chosen because it accommodates several signal program cycles, thereby enabling the agents to observe the outcomes of their actions. Thus, the action space set consists of four possible actions. Each action (i.e., signal program) is suitable for a respective part of the day or different intersection congestion level. Depending on its performance, the Q-Learning algorithm learns the control policy while trying to maximize the reward, which is calculated as the negative time loss due to vehicles driving slower than the desired speed for the case of free flow traffic. This approach incentivizes the agent to select actions that will yield a lower time-loss value.

### 4.3. Augmentation of Independent Intersection Control to Intersection Network

To apply the presented approach in a small network of intersections, each intersection has its own agent that learns a local control policy [30]. In this paper, two SOM–STM-based multi-agent approaches are compared regarding the control quality of an intersection network. The first approach uses independent agents without any incentive for cooperation. In the second approach, a reward sharing approach with a cooperation incentive is implemented by introducing a cooperation coefficient that scales the rewards from neighboring intersections before adding them to the local reward. In this research, every intersection can have one of 16 states, which is the output of the off-line trained SOM, and the state estimation is done periodically. The reward is also periodically measured as the time-loss value at the intersection inbound roads. Based on the received reward, the agents will learn which actions yield the best reward for the estimated intersection state.

#### 4.3.1. Independent Agents

The Q-Learning algorithm, where agents are independent, is the simplest approach. The agents learn independently, not knowing the states and actions of neighboring agents, and depending on its performance, each agent gets a locally calculated reward as follows:

(7) $$r_{L}\left(n\right)=-timeLoss_{t},$$

where $timeLoss_{t}$ is the calculated time lost on observed transitions in the current time step.

The independent agent approach has the advantage that the system is easy to set up. If one intersection is out of function (e.g., traffic light malfunction), the rest of the system operates independently. Each intersection is controlled by a single agent, and the traffic flow output of neighboring intersections is the traffic flow input to the observed intersection. During the operation, respective agents are receiving rewards based on their actions, and the goal of every agent is to maximize its own reward. An agent’s actions can yield a good reward, but they can generate problems at a neighboring intersection. It can happen that the agent on the neighboring intersection receives a bad reward no matter which action it selects. If such a scenario happens, the agents cannot learn the optimal control policy because their actions were not rewarded accordingly. Such a worst-case scenario is related to the case of severe traffic congestion in the controlled small intersection network.

#### 4.3.2. Reward Sharing

A multi-agent Q-Learning algorithm with reward sharing enables cooperation between neighboring agents. The benefit of this approach is the agent cooperation. The drawback is introducing an additional parameter, which increases system complexity. This approach calculates the reward as time lost on the observed transition in the current time step. Each agent gets its locally calculated reward as follows:

(8) $$r_{A_{1}}\left(t\right)=r_{L_{1}}+kr_{A_{2_{L}}},$$

(9) $$r_{A_{2}}\left(t\right)=r_{L_{2}}+\frac{k}{2}\left(r_{A_{1_{L}}}+r_{A_{3_{L}}}\right),$$

(10) $$r_{A_{3}}\left(t\right)=r_{L_{3}}+kr_{A_{1L}},$$

where $r_{A_{i}}$ denotes the total reward, $r_{L_{i}}$ denotes the local reward for each agent, *k* is the parameter that defines the intensity of influence of the neighboring agent, and $r_{A_{i_{L}}}$ denotes the local reward that the neighboring agent received.

Reward sharing encourages agents not to use actions that will significantly harm the agents on the neighboring intersections; thus, cooperation is achieved. The problem with this is that the agent can receive a bad reward because of the neighbor agent’s negative influence without having done anything wrong. The reward-sharing RL approach also has the phenomenon of freeloading, when agents participating in shared reward schemes exploit the cooperative efforts of neighboring agents without contributing proportionally to the overall objective. Such a situation can affect learning dynamics and result in suboptimal system performance. Since in this approach the agents share their rewards, the intersections are mutually dependent. In case one of the agents is inoperative, the control system will not perform as designed. Thus, these kinds of systems require a fallback safety to be implemented.

Tuning the *k* parameter value adds another layer of complexity since it determines the level of cooperation. If the *k* value is too high, it can happen that the agents tolerate a low local reward because of the neighboring agent performing well, as observed in the paper [24]. The authors selected k=0.75, noting that cooperation contributes only if there is good insight into the state of the traffic network. In [31], the authors noted that neither fully cooperative (k=1) nor fully non-cooperative (k=0) scenarios produce the best results, while k=0.5 produced the best result. The authors even observed that the k-value depends on the traffic demand, suggesting that a dynamic k-value would be ideal. The authors of [32] tested cooperative agents with k=0.5 and k=1 cooperation levels, where obtained results indicated that the k=0.5 setting performs better compared to higher k-value.

## 5. Results

This section presents the results obtained with the proposed RL-based ATSC described in Section 4, where two different ATSC approaches were implemented and compared with the FTSC approach. Measured values are collected on the microscopic level from each CV with 1[s] resolution. Collected data are then mapped to the corresponding intersection approach, and aggregated into 5-min intervals.

### 5.1. Simulation Framework

**Figure 4:**
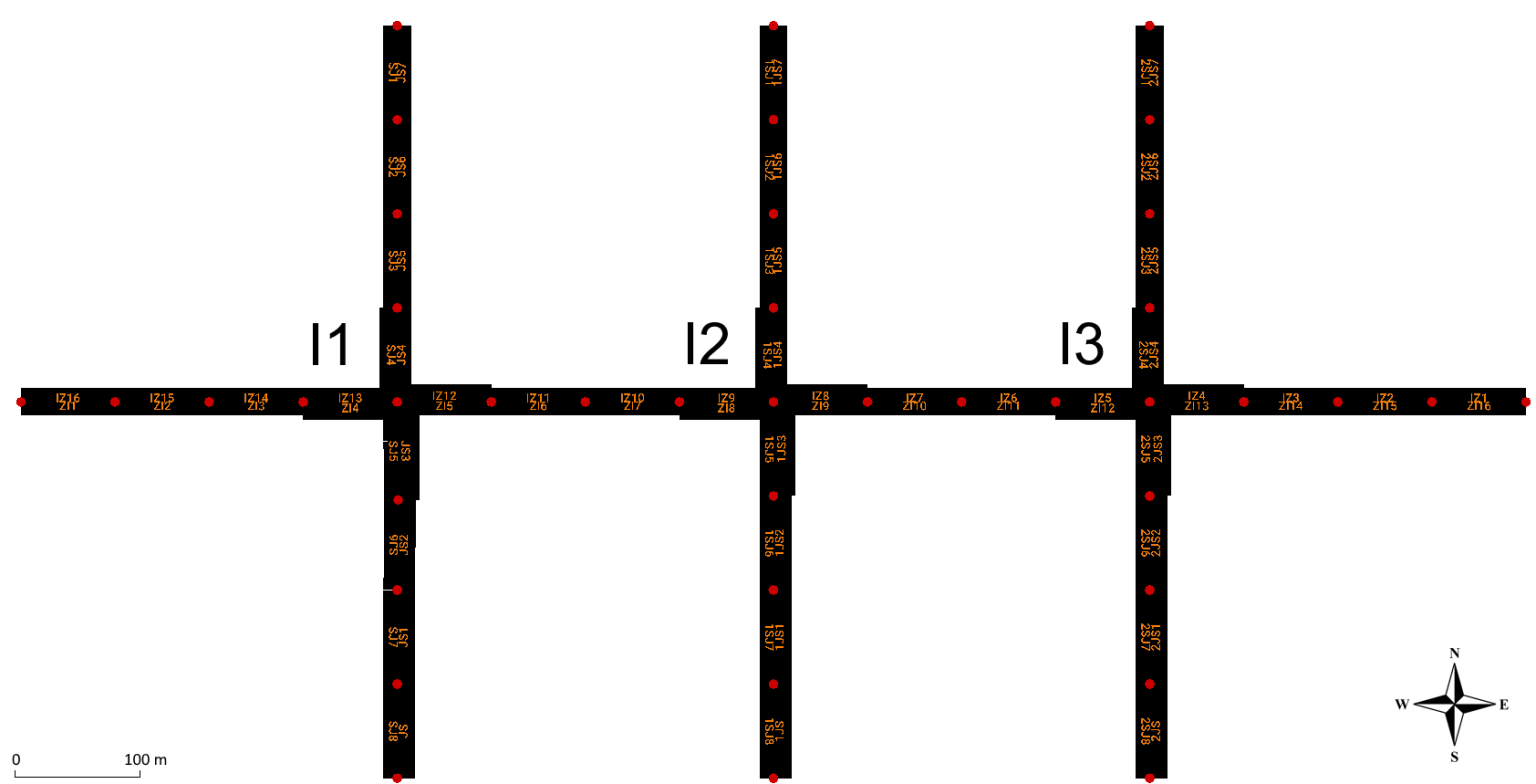
Configuration of the intersection network in the used simulation model.

**Figure 5:**
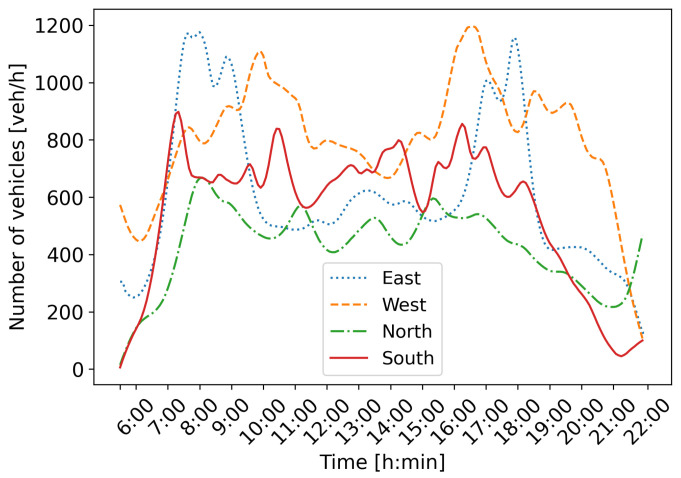
Traffic demand at the intersection of King Zvonimir Street and Heinzelova Street.

For this research, the open-source software Simulation for Urban MObility (SUMO) version 1.19.0 [33], combined with the synthetic simulation model shown in Figure 4, is used. The used synthetic simulation model is inspired by the realistic model of the intersection of King Zvonimir Street and Heinzelova Street used in our previous research [5,6]. This intersection is part of the Croatian City of Zagreb’s important urban arterial corridor and represents an average working day from 5:30 to 22:00. Traffic demand for each direction is shown in Figure 5.

It can be observed that directions east and west have more pronounced morning and afternoon peak hours. Directions north and south do not have such pronounced peak hours, but the traffic varies during the day. The speed limit is set to 50 km/h, and the intersection operates in the FTSC regime with four signal programs scheduled to activate during the day. Signal programs are activated during an apriori set time period, regardless of traffic state at the intersection. These signal programs are used as actions in our MARL-based ATSC approach.

The synthetic simulation model shown in Figure 4 is designed with the goal of testing the proposed MARL-based ATSC, where each agent is tasked to control one intersection. Each intersection has four different signal programs that are scheduled to change during the day, and each agent can select one of the existing signal programs for a particular action. Due to the limited traffic data available and the specificity of the proposed method, a synthetic model consisting of three connected consecutive intersections was created for the proof of concept. According to graph theory, three consecutive intersections form a small intersection network [34]. This model facilitates the interpretation of results and the detection of possible issues.

As mentioned, the simulation used the default car-following model with the lcKeepRight and sigma settings adopted to adjust to the local vehicle behavior. The lcKeepRight setting defines the eagerness for following the obligation to keep right. This value is set to 0.5 to force the vehicles to use more lanes, instead of just following the right lane until they have to turn left or right. The Sigma parameter is also set to 0.5. This parameter defines the driver imperfection, where the value 0 denotes perfect driving; thus, the value 0.5 is chosen to simulate human driver behavior.

The simulation model used in this research is a synthetic small network of intersections developed based on a realistic reference scenario, primarily intended to provide proof of concept for the proposed STM-based MARL framework. Thus, standard accuracy metrics concerning real-world traffic data are not directly applicable, because the network and vehicle flows were designed synthetically rather than calibrated against the on-site measurements. Nevertheless, the model parameters (e.g., car-following, lane-changing, and driver behavior settings) were selected to reflect realistic vehicle behavior, ensuring that the simulation results provide meaningful insights into the performance of the proposed approach.

### 5.2. Cooperation Parameter Sensitivity Analysis

**Figure 6:**
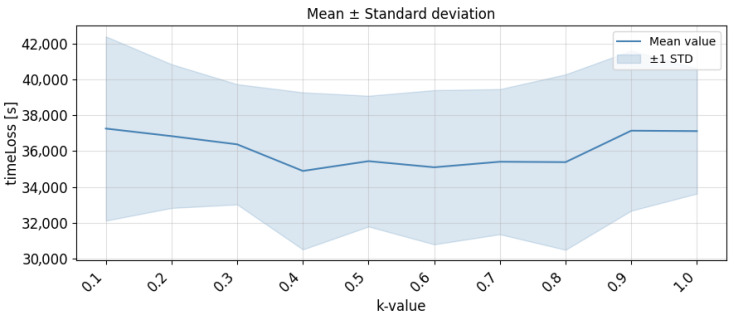
Graph of timeLoss value for k ranging from 0.1 to 1.0.

Figure 6 shows the mean value and standard deviation of the timeLoss parameter for the k-values ranging from 0.1 to 1.0 in increments of 0.1. It can be observed that the k value 0.4 has the lowest mean value. The next lowest mean value is observed with k=0.6, while k=0.5 has a higher mean value compared to 0.4 and 0.6, but the standard deviation is lower, which indicates that the implemented MARL-based ATSC is more stable.

The results of the cooperation parameter k sensitivity analysis are shown in Table 1. From the obtained results, it can be observed that k=0.4 provides the lowest μ timeLoss value with the σ value 4387.03[s]. Although the μ value for k=0.4 is the lowest, k=0.5 provides a lower σ value of 3650.87[s], which indicates a more stable traffic control system.

### 5.3. Obtained Results

This section presents the obtained results given for each intersection separately, and an overall overview of the controlled intersection network performance. The presented results are obtained after 500 simulations of training. Performance is measured as timeLoss and queue length [m], obtained from the SUMO simulator through the traCI interface. The timeLoss parameter measures how much time the vehicle lost due to driving slower than desired and is measured in seconds [s].

**Figure 7:**
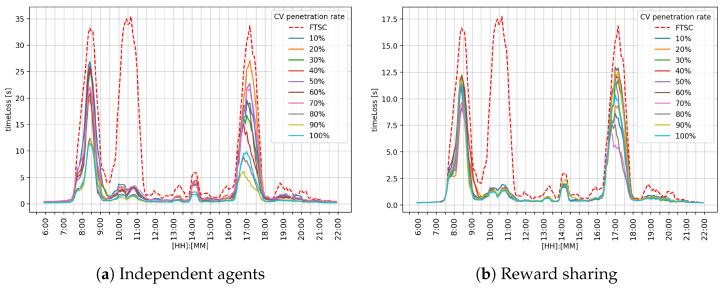
Graphical comparison of the timeLoss on the intersection $I_{1}$.

Figure 7 shows the obtained results for the intersection $I_{1}$ for both MARL approaches: the reward sharing approach presented in Figure 7a, and the reward sharing approach presented in the Figure 7b. The *x*-axis denotes the time of the day, and the *y*-axis denotes timeLoss value in seconds. At this intersection, it can be observed that FTSC is outperformed by the RL agents. Compared to independent agents and FTSC, reward sharing achieved better performance during the morning and afternoon peak hours.

Performance analysis for the different cooperation strategies is shown in Table 2. It can be observed that the reward-sharing approach outperformed FTSC as well as independent agents. Reward sharing achieved 1.2 [s] mean (μ) timeLoss, standard deviation (σ) 1.91 [s], and max value 10.56 [s]. Independent agents also reduced the mean timeLoss value to 1.75 [s], σ value to 3.30 [s], and max value to 16.59 [s]. Independent agents improved performance, but the standard deviation indicates performance inconsistency. Compared to FTSC and independent agents, the reward-sharing method shows the best overall efficiency and stability in reducing lost time, which makes it the most reliable approach of the three analyzed.

The results obtained for the independent agents approach regarding intersection $I_{1}$ are presented in Table 2. Results indicate that the independent agent approach achieves improvement over the FTSC, with the mean value of the parameter timeLoss decreasing as the CV penetration rate increases. Although the timeLoss decreases, σ remains relatively high, indicating the lower stability of the MARL system.

Table 3 shows the results of the reward-sharing approach for intersection $I_{1}$. The obtained results indicate significantly better performance compared to the independent agents and the FTSC system. The σ value tends to decrease as the CV penetration rate increases. The standard deviations are lower in all phases than in the independent agents approach, indicating better stability and predictability of the MARL system behavior. These results show that reward sharing encourages cooperative behavior among agents, which results in reduced timeLoss.

**Figure 8:**
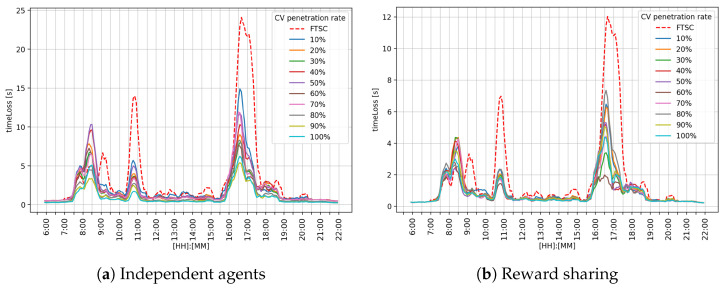
Graphical comparison of the timeLoss on the intersection $I_{2}$.

A graphical comparison of the performance metrics on the middle intersection $I_{2}$ is shown in Figure 8. It can be observed that during the morning peak hour, reward sharing achieved better performance, as shown in Figure 8b, while the independent agents approach performed close to FTSC, as shown in Figure 8a. During afternoon peak hour, both the reward sharing and independent agents achieved better performance compared to the FTSC approach.

Performance analysis for the independent agents approach is shown in Table 4. It can be observed that independent agents outperformed FTSC. The obtained results indicate that the μ value tends to decrease as the CV penetration rate increases. However, σ values show that the system has some variability in its performance, indicating lower stability of the MARL system.

Performance analysis for the shared approach is shown in Table 5. It can be observed that reward sharing outperformed FTSC. Compared to the independent approach, reward sharing achieved even better performance. The obtained results indicate that the μ value tends to decrease as the CV penetration rate increases. However, σ values are lower compared to FTSC and the independent agents approach, which indicates higher stability of the MARL system.

**Figure 9:**
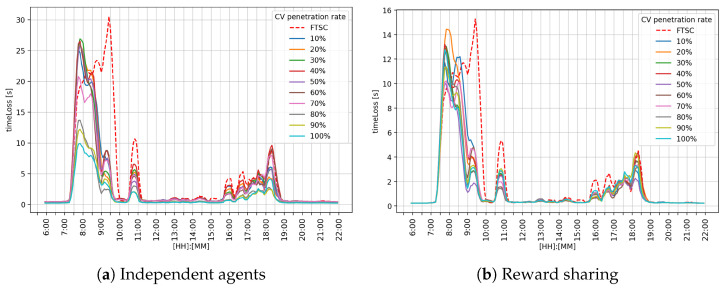
Graphical comparison of the timeLoss on the intersection $I_{3}$.

Figure 9 shows a graphical representation of the achieved timeLoss at the intersection $I_{3}$. The obtained results indicate that the reward-sharing approach shown in Figure 9b achieved better performance compared to FTSC and independent agents, as shown in Figure 9a. During afternoon hours, both MARL ATSC approaches performed better than FTSC.

A performance analysis for the independent agents approach at the intersection $I_{3}$ is shown in Table 6. Obtained results show that the μ value decreases as the CV penetration rate increases, while σ indicates that the MARL system has notable variability in its performance.

A performance analysis for the reward-sharing approach at the intersection $I_{3}$ is shown in Table 7. Compared to FTSC and the independent agents approach, this approach achieved even better results. Although the μ value does not decrease significantly, the reward-sharing approach has better stability, which can be observed from σ values that are very consistent throughout CV penetration rate range.

**Figure 10:**
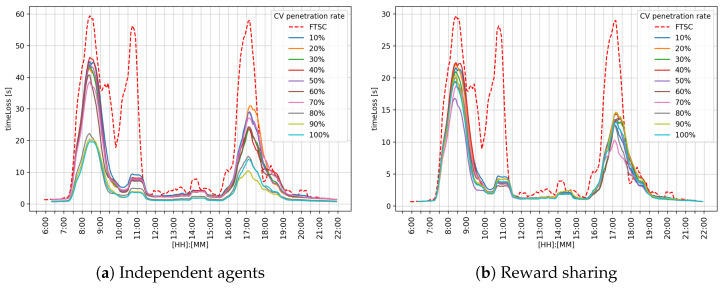
Graphical comparison of the overall timeLoss on the overall network.

Figure 10 shows the overall performance for the intersection network. The graph in Figure 10 shows that the reward-sharing approach shown in Figure 10b achieved better results than FTSC and independent agents, as shown in Figure 10a. Both independent agents and the reward-sharing approach alleviated the morning and afternoon peak hours. The most significant difference is a reduction in the peak that appears around 10:30, where MARL ATSC systems have almost eliminated it.

Table 8 shows an analysis of the overall performance of the independent agents approach for the analyzed intersection network. The obtained results indicate that the μ value decreases as the CV penetration rate increases, with a significantly better result achieved above the 20% penetration rate. Relatively high σ values indicate that the ATSC system is not very stable.

Table 9 shows an analysis of the overall performance of the independent agents approach for the analyzed intersection network. The obtained results indicate that the μ value decreases as the CV penetration rate increases, with a significantly better result achieved above the 20% penetration rate. Relatively high σ values indicate that the ATSC system is not very stable.

The influence of the CV penetration rate was tested on 500 simulations, and timeLoss and queue length parameters were selected as performance metrics. A setting of 500 simulations is selected because cooperation parameter *k* showed the greatest impact on the learning system after 500 simulations, when the RL system already converged.

#### 5.3.1. Analysis of the TimeLoss Parameter

**Figure 11:**
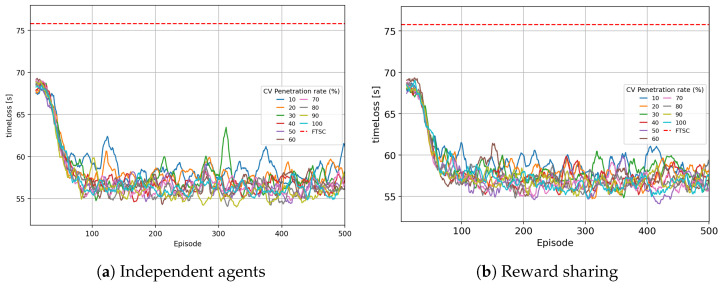
Side by side comparison of timeLoss results over 500 simulations.

Figure 11 shows a comparison of the timeLoss parameter across learning episodes for two MARL approaches: Figure 11a, a system with independent agents, and Figure 11b, a system with reward sharing. In both cases, the impact of different CV penetration rates is observed, with the dashed red line being a reference representing the performance of conventional FTSC. In both approaches, a rapid decrease in timeLoss is observed during the first 100 episodes, after which the system reaches a steady state. However, larger oscillations are observed between episodes and between different CV penetration rates in the independent agents approach, as shown in Figure 11a, indicating greater variability in the behavior of agents learning independently.

In the case of a reward-sharing system, as shown in Figure 11a, oscillations between episodes are less pronounced; thus, the system shows more robust behavior at different CV penetration rates. These results indicate that the introduction of the reward-sharing mechanism contributes to a more efficient learning process and more coordinated traffic control, thus achieving more stable and reliable TSC compared to the independent agents approach.

Table 10 presents the descriptive statistics of the timeLoss parameter for the independent agents approach. The results are collected for different CV penetration rates ranging from 10% to 100% and for FTSC without CVs. For the independent agents approach, the μ values have a reducing trend as the CV penetration rate increases. The results show moderate σ values, which indicates the presence of variability in the ATSC system.

The results obtained for the reward-sharing approach are presented in Table 11, where μ timeLoss values are reduced above a 50% CV penetration rate, while the σ remain consistently low towards the end of the penetration range, confirming the improved robustness of the system introduced with reward sharing between the agents.

Thus, while the reward-sharing approach does not substantially reduce the average timeLoss compared to independent agents, it improves system stability and robustness by reducing performance fluctuations across episodes and CV penetration rates.

#### 5.3.2. Analysis of the Queue Lengths

Table 12 shows descriptive statistics of queue length for the independent agents approach. Analysis of the queue length parameter for the independent agents approach at the intersection $I_{1}$ shows that the queue length tends to decrease as the CV penetration rate increases, with the lowest values at the 80% CV penetration rate. However, the σ indicates variability between episodes, suggesting the sensitivity of the system to intersection network dynamics. Thus, the decreasing trend of queue length indicates that a higher CV penetration rate can contribute to a better performance of the MARL ATSC.

Table 13 shows the results of the queue length for the reward-sharing approach. Analysis shows that the queue length tends to decrease as the CV penetration rate increases, with the lowest values at the 70% CV penetration rate. However, the σ indicates less variability between episodes compared to the independent agents approach, suggesting that the shared learning approach is more robust to variations in the traffic demand, which is expected.

Results of queue length on intersection $I_{2}$ for the independent agents learning approach are presented in Table 14. The lowest values are reached at the 70% CV penetration rate. However, σ values indicate variability in the system performance. This approach successfully reduces queue lengths compared to FTSC but shows limitations in the performance consistency over different penetration rates.

Table 15 shows the results of the reward-sharing approach for the same intersection $I_{2}$. Compared to the independent agents, the reward-sharing approach shows more uniform and stable results. The μ values are significantly lower than the FTSC reference value, with a slight improvement trend towards the end of the CV penetration range. The standard deviations are low for all analyzed penetration rates, indicating better stability of the ATSC system.

Table 16 shows the results of the queue length at the intersection $I_{3}$ for the independent agents approach. Compared to the FTSC, the independent agents approach achieves a significant reduction in queue length. The lowest values are recorded around 50–80% penetration rate, when the system achieves the lowest queue length and σ values. However, slight variability towards the end of penetration range indicates that the system occasionally deviates from the optimum, which may be due to a lack of coordination among the agents.

Table 17 shows the results of the queue length at the intersection $I_{3}$ for the independent agents approach. Compared to the FTSC, the independent agents approach achieves a significant reduction in queue length. The lowest values are recorded around 50–80% penetration rate, when the system achieves the lowest queue length and σ values. However, slight variability towards the end of penetration range indicates that the system occasionally deviates from the optimum, which may be due to a lack of coordination among the agents.

#### 5.3.3. Seed Analysis

Seed analysis is conducted by generating 10 new seeds for each penetration rate ranging from 10% to 100%. Analysis is conducted for the independent agents approach as well as the reward-sharing approach. Table 18 shows an analysis of the timeLoss parameter for different seeds for the independent agents approach. Each penetration rate is tested with 10 different seeds on MARL ATSC. The obtained results show that different seeds have no significant influence on the MARL ATSC system performance. The obtained results indicate that the μ value of timeLoss is reduced as the CV penetration rate increases.

Table 18 shows that the μ value tends to decrease as the CV penetration rate increases, with the best results towards the end of CV penetration range. The decreasing μ value with relatively small σ value indicates that the independent agents approach is robust to unseen scenarios of different seeds.

Table 19 shows descriptive statistics of the timeLoss parameter for different seeds on the reward-sharing approach. Obtained results indicate that the ATSC system has the best performance around 60% CV penetration rate. The σ value is relatively small, indicating that the shared learning approach is robust to variations in the traffic demand of different seeds.

The analysis shows that the reward-sharing approach ensures relatively stable performance, with little oscillations in performance. Although the μ values vary across different CV penetration rates, the variation is generally small, suggesting robustness of the MARL ATSC and robustness to new scenarios.

## 6. Discussion

The results presented in Section 5 are collected in the simulation of the modeled small network of intersections. The obtained results include high-resolution vehicle speeds data obtained from CVs in a microscopic simulation environment. The purpose of the research was to develop and test an RL-based ATSC system that uses STM for traffic state estimation. Results were obtained at the intersection inbound (approaching road) segments on three consecutive intersections. For each intersection, traffic state is estimated based on collected CV data, and the estimated state is further used as input for the MARL-based ATSC system, where each intersection is controlled by one corresponding agent. Independent agents and cooperative agents were tested and compared to the FTSC. Analysis is conducted for each intersection independently and for the complete network as a global overview.

Observing the performance metrics (timeLoss and queue length) for each intersection individually, it can be seen that the reward-sharing learning approach achieves better results during the morning and afternoon peak hours. Such an outcome is expected for a cooperative system. The reason for such behavior could be the impact of the k-value, which defines the level of influence between neighboring agents. Independent agents followed the greedy policy, trying to maximize their own reward, while cooperative agents shared their rewards. That means the agent could make a bad action and still receive a good reward because the neighboring agent selected a good action. In overall performance comparison, reward sharing achieved a better μ value of timeLoss and queue length. In graphical representation, regarding Figure 7, Figure 8 and Figure 9, it can be observed that reward sharing struggles for a short period of time with the morning peak hour, and after that, outperforms other control approaches. The σ value is higher compared to independent agents, which means the agent’s actions have more influence on the timeLoss. These results indicate that the reward-sharing approach has potential but requires additional tuning. A reduction in timeLoss indicates that vehicles are traveling closer to their desired speed (i.e., the speed limit). Thus, if the speed distribution were represented in the STM, it would tend to cluster toward the fourth quadrant, reflecting improved traffic flow efficiency.

The cooperation between agents is defined by a tunable parameter k, which defines the level of influence of agents on each other. The system is tested over a broad range of k-values ranging from 0.1 to 1.0 to determine its sensitivity and optimal performance limits. Results indicated that k = 0.5 value achieved the best performance with the lowest timeLoss value. Such results aligns with other research results [24,31,32], where it is observed that overly strong mutual influence between agents is counterproductive. While previous research has analyzed the impact of CV penetration rate on information quality, its direct impact on the performance of RL-based ATSC systems has not yet been fully explored. Analysis of the different seeds across the CV penetration range from 10% to 100% indicates that both approaches gain performance improvements in terms of timeLoss and queue length values as the CV penetration rate increases. As expected, the reward-sharing approach achieved better stability across the penetration range, meaning that the system is more robust to new scenarios.

Scaling the system to a larger network of intersections can be effectively achieved through a decentralized architecture, where each intersection operates as an independent agent. While a decentralized approach significantly improves scalability and robustness, it also introduces non-stationarity, making it more challenging for coordinated learning to achieve global objectives. In terms of agent coordination, the simplest approach would involve sharing information only with neighboring agents. Neighbor-to-neighbor coordination is a practical and scalable approach, especially in real-world urban environments where communication and computational resources are limited.

## 7. Conclusions

This paper presented a MARL-based ATSC system, which uses STMs for the traffic state estimation of a small network of signalized intersections. The proposed ATSC system is developed and tested in a microscopic simulation environment and compared to an FTSC baseline, as well as two RL-based approaches: independent agents and reward sharing.

Obtained results show that the reward-sharing approach achieves the lowest average values of timeLoss and queue length values across the tested scenarios. In addition, the value of the standard deviation indicates a higher variability of the behavior of cooperative agents, which may be the result of reward propagation between agents. Such behavior indicates that the agents cooperate through the reward-sharing approach. Examining the sensitivity of the system to different levels of cooperation, the results indicated that a cooperation level of k=0.5 achieves the best ATSC performance. Seed analysis showed that both approaches are capable of handling unseen scenarios. Thus, the reward sharing approach is more robust to changes in traffic demands as a result of the agents’ cooperation.

This paper focused on the learning behavior of the agents assuming reliable V2X communication, while sensor failures or communication issues were tested through reduced CV penetration rates. A detailed analysis of communication reliability and sensor degradation remain outside of the scope of this paper. Future work should research additional cooperation strategies beyond reward sharing to enable a broader performance comparison within decentralized MARL-based ATSC systems, extend the system to larger networks, and explore more advanced coordination mechanisms, focused on scalability and real-world applicability.

## Figures and Tables

**Table 1:** Analysis of the cooperation parameter (*k*) value.

k	0.1	0.2	0.3	0.4	0.5	0.6	0.7	0.8	0.9	1.0
μ	37,254.99	36,832.57	36,373.82	34,888.91	35,435.06	35,093.44	35,403.37	35,380.64	37,136.84	37,113.56
σ	5144.08	4013.65	3357.55	4387.03	3650.87	4306.02	4052.44	4899.34	4470.94	3493.80

**Table 2:** Analysis of the timeLoss parameter for the independent agents approach at the intersection $I_{1}$.

CV (%)	FTSC	10	20	30	40	50	60	70	80	90	100
μ	3.82	1.54	1.60	1.50	1.44	1.43	1.57	1.62	1.33	1.33	1.56
σ	5.60	3.17	3.24	3.05	3.03	3.02	3.26	3.31	2.75	2.75	3.27

**Table 3:** Analysis of the timeLoss parameter for the reward-sharing approach at the intersection $I_{1}$.

CV (%)	FTSC	10	20	30	40	50	60	70	80	90	100
μ	3.82	1.60	1.46	1.60	1.59	1.40	1.56	1.51	1.36	1.53	1.34
σ	5.60	3.24	3.01	3.28	3.37	2.85	3.22	3.03	2.81	3.06	2.80

**Table 4:** Analysis of the timeLoss parameter for the independent agents approach at the intersection $I_{2}$.

CV (%)	FTSC	10	20	30	40	50	60	70	80	90	100
μ	1.70	1.00	0.97	0.89	0.84	0.90	0.84	0.90	0.85	0.83	0.80
σ	2.75	1.59	1.59	1.36	1.28	1.44	1.25	1.41	1.28	1.27	1.20

**Table 5:** Analysis of the timeLoss parameter for the reward sharing approach at the intersection $I_{2}$.

CV (%)	FTSC	10	20	30	40	50	60	70	80	90	100
μ	1.70	1.02	1.03	0.85	0.90	0.85	0.72	0.87	1.02	0.91	0.84
σ	2.75	1.60	1.62	1.27	1.46	1.27	0.87	1.35	1.62	1.37	1.29

**Table 6:** Descriptive statistics of timeLoss parameter for the independent agents approach at the intersection $I_{3}$.

CV (%)	FTSC	10	20	30	40	50	60	70	80	90	100
μ	2.28	1.59	1.58	1.57	1.56	1.46	1.37	1.41	1.47	1.47	1.35
σ	3.89	3.17	3.22	3.25	3.16	2.98	2.96	2.71	2.91	2.85	2.90

**Table 7:** Descriptive statistics of timeLoss parameter for the reward-sharing approach at the intersection $I_{3}$.

CV (%)	FTSC	10	20	30	40	50	60	70	80	90	100
μ	2.28	1.68	1.70	1.43	1.65	1.22	1.42	1.49	1.43	1.45	1.45
σ	3.89	3.52	3.49	2.91	3.24	2.35	2.85	2.96	2.78	2.77	2.83

**Table 8:** Descriptive statistics of timeLoss parameter for the network overall.

CV (%)	FTSC	10	20	30	40	50	60	70	80	90	100
μ	7.81	4.43	4.17	3.87	4.11	4.06	3.71	4.01	3.49	3.76	3.87
σ	9.48	6.47	6.22	5.89	6.30	6.34	5.55	5.69	5.05	5.80	5.92

**Table 9:** Descriptive statistics of timeLoss parameter for the network overall.

CV (%)	FTSC	10	20	30	40	50	60	70	80	90	100
μ	7.81	4.23	4.21	3.92	4.35	3.52	3.85	3.61	3.77	4.06	3.80
σ	9.48	6.18	6.24	5.86	6.61	5.05	5.63	5.33	5.50	5.86	5.75

**Table 10:** Analysis of the timeLoss parameter for independent agents approach.

CV (%)	10	20	30	40	50	60	70	80	90	100
μ	58.24	55.49	57.62	57.39	56.62	56.31	56.42	56.39	55.96	56.17
σ	1.67	1.44	1.64	2.11	1.44	1.62	1.57	1.51	1.61	1.48

**Table 11:** Analysis of the timeLoss parameter for the reward-sharing approach.

CV (%)	10	20	30	40	50	60	70	80	90	100
μ	58.49	56.77	57.49	57.68	57.27	56.49	56.68	56.56	56.91	56.34
σ	1.84	1.66	1.79	1.87	1.63	1.80	1.56	1.55	1.59	1.55

**Table 12:** Results of queue length on intersection $I_{1}$ for independent agents learning approach.

CV (%)	FTSC	10	20	30	40	50	60	70	80	90	100
μ	19.9	14.45	13.98	13.87	13.62	13.65	13.64	14.2	13.47	13.52	13.99
σ	-	0.51	0.47	0.49	0.3	0.46	0.3	0.75	0.28	0.48	0.74

**Table 13:** Results of queue length on intersection $I_{1}$ for reward sharing learning approach.

CV (%)	FTSC	10	20	30	40	50	60	70	80	90	100
μ	19.9	14.29	13.87	13.73	13.99	13.61	14.03	13.47	13.88	14.36	13.7
σ	-	0.38	0.49	0.34	0.52	0.37	0.61	0.42	0.55	0.8	0.42

**Table 14:** Results of queue length on intersection $I_{2}$ for independent agents learning approach.

CV (%)	FTSC	10	20	30	40	50	60	70	80	90	100
μ	20.66	12.68	11.01	9.99	10.5	12.4	10.77	10.95	12.18	11.75	10.81
σ	-	1.39	1.56	0.61	1.31	1.23	0.62	1.24	1.35	1.52	0.89

**Table 15:** Results of queue length on intersection $I_{2}$ for reward sharing learning approach.

CV (%)	FTSC	10	20	30	40	50	60	70	80	90	100
μ	20.66	11.54	11.31	10.34	10.39	10.29	10.05	10.39	12.12	11.16	9.85
σ	-	1.35	1.53	1.53	0.98	1.14	0.49	0.86	1.11	1.42	0.88

**Table 16:** Results of queue length on intersection $I_{3}$ for independent agents learning approach.

CV (%)	FTSC	10	20	30	40	50	60	70	80	90	100
μ	18.71	11.92	11.5	11.55	11.58	10.67	10.74	11.62	10.67	10.84	10.58
σ	-	1.59	1.64	1.6	1.39	1.03	1.37	1.4	0.71	1.42	1.26

**Table 17:** Results of queue length on intersection $I_{3}$ for reward-sharing learning approach.

CV (%)	FTSC	10	20	30	40	50	60	70	80	90	100
μ (m)	18.71	11.43	11.35	10.79	10.55	10.74	10.63	11.74	11.44	11.6	11.82
σ	-	1.76	1.42	1.59	1.45	1.3	1.31	1.36	1.33	1.56	1.51

**Table 18:** Analysis of different seed influence on timeLoss for independent agents approach.

%	10	20	30	40	50	60	70	80	90	100
μ	61.24	57.32	55.55	56.57	54.82	55.73	57.32	55.70	53.50	53.17
σ	0.36	0.41	0.56	0.84	1.18	0.45	0.53	0.50	0.77	0.62

**Table 19:** Analysis of different seed influence on timeLoss for reward sharing approach.

%	10	20	30	40	50	60	70	80	90	100
μ	63.17	55.88	54.53	53.53	55.78	53.46	56.3	58.33	56.69	58.46
σ	0.63	0.9	0.75	0.83	0.73	0.68	0.75	1.16	0.58	0.52

## Data Availability

The original contributions presented in this study are included in the article. Further inquiries can be directed to the corresponding author.

## Abbreviations

The following abbreviations are used in this manuscript:

ADAS	Advanced driver assistance systems
ATSC	Adaptive traffic signal control
BMU	Best matching unit
CAV	Connected autonomous vehicle
CoM	Center of mass
CV	Connected vehicle
FTSC	Fixed traffic signal control
GNG	Growing neural gas
ML	Machine learning
RL	Reinforcement learning
SOM	Self-organizing map
STM	Speed transition matrix
SUMO	Simulation for Urban MObility
TSC	Traffic signal control
V2X	Vehicle to everything
